# Non-Surgical Correction of Facial Asymmetry: A Narrative Review of Non-Surgical Modalities and Clinical Case Examples

**DOI:** 10.3390/jcm14248828

**Published:** 2025-12-13

**Authors:** Clara Lee, Sumin Chae, Han-Jin Kwon, Wonwoo Jeong, Kyung Kwan Lee, Minsuk Chae

**Affiliations:** 1 Dr. Queens Clinic, Gangnam Main Branch, Specialized in Non-Surgical Aesthetic Treatments, Seoul 06223, Republic of Korea; drqueensclinic@naver.com (C.L.); zasmin31@gmail.com (S.C.); 2 Dermaster Clinic, Seoul 06014, Republic of Korea; kohaji333333@gmail.com; 3 Wake Forest Institute for Regenerative Medicine, Wake Forest School of Medicine, Winston-Salem, NC 27157, USA; wonwoo.jeong@advocatehealth.org; 4 Department of Bio-Chemical Engineering, Chosun University, 309 Pilmun-Daero, Dong-Gu, Gwangju 61452, Republic of Korea; 5 Department of Anesthesiology and Pain Medicine, College of Medicine, The Catholic University of Korea, Seoul 06591, Republic of Korea

**Keywords:** facial asymmetry, dermal fillers, polydioxanone thread, collagen stimulator, radiofrequency, shockwave therapy

## Abstract

Facial asymmetry significantly affects aesthetic appearance, essential functions such as mastication and speech, and psychological well-being. While traditional surgical interventions effectively address significant facial asymmetry, they are often associated with considerable morbidity, prolonged recovery periods, and potential complications. Consequently, interest in minimally invasive, non-surgical techniques has substantially increased, driven by advantages including reduced downtime, rapid recovery, and immediate aesthetic results. This narrative review critically evaluates contemporary non-surgical techniques for correcting facial asymmetry, focusing specifically on dermal fillers, collagen stimulators (polydioxanone powder), polydioxanone thread lifting, energy-based non-invasive devices (radiofrequency, ultrasound, and laser therapies), and extracorporeal shockwave therapy. The review is based on a structured literature search of PubMed/MEDLINE, Embase, and Google Scholar up to October 2025, focusing on human clinical studies and review articles on non-surgical correction of facial asymmetry and related facial contouring. We provide a detailed analysis of each treatment modality’s underlying mechanisms, clinical efficacy, advantages, limitations, and safety profiles. Current evidence suggests that these non-surgical methods effectively enhance facial symmetry by offering immediate visible improvements and progressive enhancements through natural collagen regeneration, thereby significantly improving patient satisfaction and overall quality of life. Clinicians are encouraged to incorporate these versatile, minimally invasive interventions into clinical practice, carefully tailoring treatments according to individual patient characteristics and specific aesthetic goals. Further research should aim to refine existing treatment protocols, evaluate long-term efficacy and safety, and establish standardized guidelines to optimize outcomes in facial asymmetry correction.

## 1. Introduction

Facial asymmetry refers to differences in the size, shape, and position of facial structures between the left and right sides of the face, significantly impacting aesthetic appearance and essential functions such as mastication, speech, and psychological well-being [1]. While minor asymmetry is common in the general population, pronounced asymmetry can lead to noticeable facial imbalance, adversely affecting an individual’s self-esteem and social interactions [2]. Epidemiological studies have reported that clinically significant facial asymmetry affects approximately 12% to 37% of orthodontic patients in the United States, with prevalence rates varying based on diagnostic criteria and assessment methods [3,4]. In South Korea, recent research similarly demonstrated a notable prevalence rate, with approximately 11.0% of orthodontic patients exhibiting clinically relevant facial asymmetry based on cephalometric evaluations [5]. Notably, when radiographic evaluations are employed, the prevalence of facial asymmetry can exceed 50%, suggesting that asymmetry may be more common than clinically observed [6].

Facial asymmetry is multifactorial, arising from a complex interplay of genetic and congenital factors, developmental influences, environmental exposures, and acquired conditions. Among these, habitual behaviors such as unilateral chewing, sleeping predominantly on one side, and prolonged use of unilateral facial muscles are commonly cited etiological contributors [4,7,8,9,10]. Malocclusion, characterized by misalignment of the dental arches, has been extensively studied in relation to facial asymmetry. A systematic review indicated that early intervention in cases of malocclusion can lead to improvements in facial symmetry, particularly in the lower face, emphasizing the importance of timely corrective measures during developmental phases. Trauma, including fractures or soft-tissue injuries, can further exacerbate facial asymmetry by disrupting normal anatomical alignment and healing processes. For instance, zygomatic fractures can lead to noticeable facial asymmetry and functional impairments if not properly addressed [11,12]. A recent systematic review emphasized that untreated malocclusion during developmental phases could progressively worsen facial asymmetry, highlighting the importance of early intervention and corrective measures [13,14].

Traditionally, surgical interventions such as orthodontics and orthognathic surgery have been standard modalities for correcting facial asymmetry [15]. Orthognathic surgery, which involves repositioning of jawbones, is effective in severe cases but is associated with significant morbidity, extensive recovery periods, and potential complications such as nerve damage, infection, and relapse [16]. Additionally, orthodontic treatments require prolonged use of corrective appliances, and patient compliance can often be challenging, leading to suboptimal results [17,18].

Considering these limitations, interest in non-surgical interventions has substantially increased in recent years. Non-surgical techniques offer multiple advantages, including minimally invasive procedures, reduced recovery times, and immediate aesthetic outcomes with lower risks of complications [19,20]. Emerging literature supports the effectiveness of non-surgical methods such as injectable dermal fillers, collagen stimulators, Polydioxanone (PDO) thread lifting, energy-based non-invasive devices (radiofrequency, ultrasound, laser), and extracorporeal shockwave therapy (ESWT) [21,22,23]. Recent clinical studies demonstrated promising results with these modalities, noting high patient satisfaction and sustained improvements in facial symmetry with minimal downtime [21,24].

Therefore, this narrative review aims to critically evaluate current non-surgical approaches for correcting facial asymmetry, exploring their mechanisms of action, clinical efficacy, safety profiles, and practical considerations for integration into clinical practice. This narrative synthesis aims to summarize current non-surgical modalities for facial asymmetry, outline practical considerations for selecting individualized treatment combinations based on etiologic patterns, and highlight gaps that warrant further clinical research. Throughout the manuscript, we explicitly distinguish between findings derived from published clinical studies and illustrative clinical case examples from an aesthetic clinic, so that readers can clearly separate literature-based evidence from real-world practice patterns.

### Literature Search Strategy

This narrative review was informed by a structured, but not fully systematic, search of the literature. We searched PubMed/MEDLINE, Embase, and Google Scholar for articles published up to October 2025. The following combinations of keywords and Medical Subject Headings (MeSH) terms were used: “facial asymmetry”, “non-surgical”, “noninvasive”, “dermal filler”, “hyaluronic acid”, “calcium hydroxylapatite”, “poly-L-lactic acid”, “polymethylmethacrylate”, “PDO thread”, “thread lifting”, “PDO powder”, “collagen stimulator”, “radiofrequency”, “HIFU”, “high-intensity focused ultrasound”, “laser”, and “extracorporeal shockwave therapy (ESWT)”. Boolean operators (AND/OR) were applied to combine terms related to facial asymmetry with terms describing specific treatment modalities.

We included English-language publications involving human subjects that reported on non-surgical approaches to correcting facial asymmetry or improving facial contour, including clinical trials, observational studies, case series, and review articles. Articles that focused exclusively on surgical correction (e.g., orthognathic surgery without a non-surgical component), animal-only experiments, basic science studies without clinical correlation, or aesthetic procedures unrelated to facial asymmetry were excluded. Titles and abstracts identified through the database searches were screened for relevance, and full texts were reviewed when necessary. In total, approximately 100 publications were considered most relevant and were used to inform the present narrative synthesis, which is further complemented by representative clinical cases from a high-volume aesthetic clinic in Korea.

Because of the narrative design and the heterogeneity of study designs, we did not perform a formal risk-of-bias assessment using standardized tools; instead, we qualitatively appraised methodological quality based on study design, sample size and follow-up duration, objectivity of outcome measures, and reporting of complications and limitations.

## 2. Clinical Categories of Facial Asymmetry

Facial asymmetry significantly impacts aesthetic appearance and psychological well-being. Non-surgical cosmetic interventions have become increasingly popular due to their minimally invasive nature, rapid recovery, and immediate visual improvements. The clinical management of facial asymmetry can be categorized based on the distinct clinical presentations and the corresponding non-surgical treatment options (Table 1).

### 2.1. Volume-Related Asymmetry

Volume-related facial asymmetry results from uneven distribution or depletion of soft-tissue volume in distinct facial regions, contributing to noticeable aesthetic imbalances. Common anatomical areas affected include the cheeks, lips, and tear troughs, each demonstrating unique clinical presentations. Aging and other etiological factors often lead to diminished subcutaneous adipose tissue and underlying skeletal remodeling in the midface region. Such changes cause flattening or hollowing of the cheeks, altering the natural midface convexity, and resulting in an aged or fatigued appearance. Restoration of midfacial volume is critical for reestablishing youthful facial contours and aesthetic harmony [25,26,27]. Lip asymmetry typically emerges due to congenital variations, trauma, or age-associated volume loss. Thinning and asymmetry of the lips and associated perioral lines significantly impact facial balance, contributing to aesthetic dissatisfaction. Restoring symmetrical lip volume is fundamental to achieving balanced facial proportions and improved aesthetic outcomes [28,29]. Volume deficiency within the tear trough area, extending from the medial canthus downward and outward, manifests as noticeable infraorbital hollowing. Clinically, this creates shadowing and perceived dark circles or under-eye bags, leading to a persistently tired and prematurely aged appearance. Addressing volume depletion in the tear trough region effectively rejuvenates the infraorbital area, markedly enhancing facial aesthetics [30].

### 2.2. Soft Tissue Sagging and Skin Laxity

Soft tissue sagging and skin laxity represent common yet prominent manifestations of facial aging, resulting from intrinsic and extrinsic factors, including genetic predisposition, cumulative sun exposure, collagen and elastin degradation, and gravitational influences. Clinically, these conditions manifest notably as drooping eyebrows, accentuated nasolabial folds, and the development of irregular jawlines or jowls. The periorbital region, particularly the forehead and eyebrows, is highly susceptible to age-related ptosis. Chronic exposure to ultraviolet (UV) radiation accelerates dermal elastosis, degrading collagen and elastin fibers, leading to loss of structural support. Consequently, the eyebrows descend, creating a persistent appearance of fatigue or sternness and exacerbating upper eyelid hooding [31,32]. Nasolabial folds, the prominent creases extending from the lateral nasal alae to the oral commissures, become more pronounced with age due to the downward migration of midface fat pads, combined with reductions in dermal collagen and elastin. Gravitational forces further amplify soft-tissue ptosis, deepening the nasolabial folds and significantly impacting facial aesthetics by imparting an aged and tired appearance [33,34]. Sagging of the lower facial soft tissues and skin laxity significantly diminishes jawline definition, forming jowls and soft-tissue protrusions along the mandibular border. This phenomenon primarily results from attenuation of ligamentous structures, subcutaneous fat displacement, and dermal collagen depletion. Progressive soft tissue laxity disturbs the youthful contour of the mandibular line, contributing substantially to an aged facial profile [35,36].

### 2.3. Dynamic (Muscular) Asymmetry

Dynamic asymmetry refers to the imbalance in facial muscle activity, manifesting predominantly as uneven facial expressions and asymmetric smiles. This asymmetry arises primarily from disruptions in neuromuscular coordination and facial nerve (cranial nerve VII) integrity, which is crucial for symmetric muscular activation. A common cause of dynamic asymmetry is facial nerve paralysis, notably seen in conditions such as Bell’s palsy, where unilateral nerve impairment leads to muscular weakness or paralysis, significantly affecting facial symmetry during expressive movements [37]. Furthermore, aberrant nerve regeneration following nerve injury may result in synkinesis, unintentional co-activation of muscles producing involuntary movements during voluntary facial actions, thereby exacerbating dynamic asymmetry [38]. Additionally, congenital muscular or neuromuscular developmental anomalies can inherently predispose individuals to lifelong facial muscle imbalances [39,40]. Clinically, affected individuals typically demonstrate noticeable asymmetry when smiling, involuntary muscle contractions during expression, and potential functional impairments impacting speech, mastication, and eye closure, thereby highlighting the significant aesthetic and functional implications of dynamic facial asymmetry [38].

### 2.4. Superficial Skin Texture Irregularities

Superficial skin texture irregularities, such as fine lines, acne scars, and uneven skin tone, significantly impact aesthetic perception by diminishing skin smoothness and radiance. These imperfections can result from factors like aging, sun exposure, and previous inflammatory skin conditions, notably acne. The breakdown of collagen and elastin due to UV damage contributes to wrinkle formation and textural roughness. Additionally, the skin’s natural aging process leads to decreased cell turnover, resulting in a dull and uneven complexion. Addressing these concerns is vital in aesthetic dermatology to enhance skin appearance and patient confidence. Treatment modalities include chemical peels, microneedling, laser resurfacing, and topical retinoids, all aimed at promoting collagen production and accelerating epidermal renewal to improve skin texture and tone [41,42].

### 2.5. Composite (Mixed) Asymmetry

Composite (mixed) asymmetry involves the simultaneous occurrence of multiple factors, including volume loss, skin laxity, muscular imbalances, and superficial skin irregularities, collectively contributing to facial aesthetic disruption. This multifaceted presentation commonly arises from aging processes, congenital conditions, or acquired structural alterations. Volume depletion significantly impacts midface contours and soft-tissue positioning, exacerbating gravitational descent and the prominence of skin laxity, thus producing irregular jawlines and deepened nasolabial folds. Concurrently, muscular imbalances introduce dynamic asymmetry through inconsistent facial expressions and movements. Additionally, superficial skin irregularities such as wrinkles, scars, and uneven textures further compound aesthetic challenges, emphasizing the complex and integrative nature of composite facial asymmetry [42,43,44].

## 3. Non-Surgical Aesthetic Modalities

**Figure 1:**
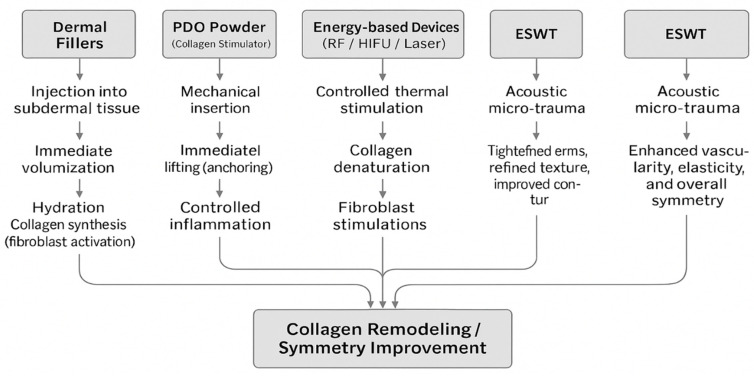
Schematic representation of the principal biological mechanisms underlying major non-surgical modalities for facial asymmetry correction.

Non-surgical aesthetic modalities have increasingly gained popularity as minimally invasive solutions to facial asymmetry, offering patients rapid recovery, immediate cosmetic improvements, and reduced complication risks compared to surgical interventions. This section provides an overview of prominent non-surgical cosmetic approaches, highlighting their key mechanisms of action, clinical applications, and associated aesthetic outcomes (Table 2). The underlying biological mechanisms of these modalities are summarized in Figure 1.

In the following subsections, the descriptions of mechanisms, efficacy, and safety are primarily based on published clinical studies, whereas our own clinical experience is presented separately as clearly labeled “representative clinical case” examples and brief “in our clinical practice” statements.

### 3.1. Dermal Fillers

#### 3.1.1. Mechanism and Applications

In published clinical studies, dermal fillers and injectable biostimulators are gel-like substances that are administered beneath the skin to restore lost volume, smooth wrinkles, and enhance facial contours. Commonly used materials include hyaluronic acid (HA), calcium hydroxylapatite (CaHA), poly-L-lactic acid (PLLA), and polymethylmethacrylate (PMMA). HA primarily provides immediate volumization by occupying space and attracting water molecules in the extracellular matrix. In contrast, CaHA, PLLA, and PMMA additionally act as potent biostimulators by inducing fibroblast activation, neocollagenesis, and long-term tissue remodeling, which can lead to progressive improvement in facial symmetry and contour over time [45,46,47,48].

Upon administration, HA fillers exhibit pronounced hydrophilic properties, attracting water molecules to the treated area. This interaction results in immediate volumetric enhancement and hydration, effectively addressing facial asymmetry by filling depressions, supporting soft tissue, and providing structural integrity to facial contours. HA’s capacity to bind water molecules over 1000 times its own weight contributes significantly to these aesthetic improvements. The efficacy of HA fillers in enhancing facial aesthetics and symmetry has been extensively documented in clinical studies. For instance, a study evaluating a resilient HA filler demonstrated significant improvement in wrinkle severity scores over a 15-month period, with notable effects observed as early as 24 weeks post-treatment. Moreover, the duration of HA filler effects varies depending on the specific product used and the anatomical region treated, typically lasting between 6 to 24 months [49,50,51,52,53].

CaHA, another widely utilized dermal filler, comprises biocompatible synthetic microspheres suspended in an aqueous gel carrier. Its mechanism of action involves an initial phase of immediate volumization, achieved by the gel component, followed by sustained biostimulatory effects through fibroblast activation around the CaHA microspheres. This stimulation promotes significant neocollagenesis, leading to structural reinforcement, enhanced tissue elasticity, and progressive improvement in dermal quality. Clinical studies have demonstrated that CaHA fillers yield durable aesthetic results, typically persisting for approximately 12 to 18 months, with some reports indicating benefits extending up to 24 months, depending on patient factors and anatomical treatment sites. The unique dual-action mechanism of CaHA fillers renders them particularly suitable for deeper volumetric deficits, contour enhancements of the cheeks and jawline, and correction of pronounced facial asymmetry. This long-lasting efficacy underscores their advantageous role in comprehensive non-surgical facial rejuvenation protocols [54,55,56].

PLLA is a biodegradable synthetic polymer extensively utilized in aesthetic medicine due to its unique biostimulatory properties, primarily inducing gradual collagen synthesis rather than immediate volumization. Upon injection, PLLA microparticles trigger a controlled inflammatory response that activates fibroblasts, leading to progressive collagen production and subsequent restoration of facial volume and symmetry over multiple treatment sessions. Clinical studies demonstrate that significant volumetric improvements become evident progressively within several weeks to months, typically requiring 2–4 sessions spaced approximately 4–6 weeks apart, with aesthetic results sustained up to approximately 24 months post-treatment. PLLA fillers are thus particularly suitable for addressing substantial volume deficits, deep facial wrinkles, contour irregularities, and structural asymmetries, providing natural-looking and durable facial rejuvenation outcomes [57,58,59,60].

PMMA fillers, such as Artefill, consist of approximately 20% non-resorbable PMMA microspheres suspended in 80% purified bovine collagen gel. Upon injection, the collagen gel provides immediate volume, while the PMMA microspheres serve as a scaffold for long-term tissue integration and neocollagenesis, resulting in enduring structural support. Due to the permanent nature of PMMA fillers, they necessitate meticulous injection techniques and extensive clinical expertise to minimize risks such as nodular masses, inflammation, allergic reactions, and discoloration. Therefore, patient selection and counseling are critical to ensure that individuals fully understand the permanent nature of PMMA fillers and the potential for long-term complications [61,62,63].

Dermal fillers are versatile injectables used to restore facial volume, smooth wrinkles, and enhance contours. Common application sites include the cheeks, lips, jawline, nasolabial folds, and marionette lines. The selection of filler type and injection technique is meticulously tailored to each individual’s anatomical structure and aesthetic goals to ensure optimal outcomes [64].

#### 3.1.2. Advantages and Immediate Aesthetic Outcomes

Dermal fillers present multiple clinically relevant advantages, contributing significantly to their popularity in aesthetic medicine. Procedures involving dermal fillers are minimally invasive, typically requiring only topical or local anesthesia, and are usually completed within 30 to 60 min. This short procedural duration enhances patient convenience and compliance. Immediate post-injection improvements are visually apparent, with optimal aesthetic outcomes typically emerging within several days as initial swelling and minor bruising resolve. This rapid onset of visible results is highly desirable for patients seeking prompt enhancements in facial symmetry and contour [21,65].

The minimal downtime associated with dermal filler treatments further underscores their practicality, as patients can promptly resume their normal daily activities with minimal disruption. This convenience significantly contributes to high patient satisfaction rates. Empirical evidence supports this observation, with patient-reported satisfaction frequently exceeding 90%, attributable to immediate and noticeable improvements in facial symmetry, contour enhancement, and overall facial aesthetics. Additionally, the reversible nature of certain fillers, such as HA, provides further reassurance to both clinicians and patients by allowing adjustments or corrections if desired outcomes are not initially achieved [21,66,67].

Clinicians’ ability to precisely tailor filler placement according to individual patient anatomy and aesthetic goals further amplifies treatment outcomes. This personalized approach, combined with immediate aesthetic benefits and minimal procedural risks, substantiates the ongoing preference for dermal fillers in non-surgical facial rejuvenation practices [21].

#### 3.1.3. Potential Drawbacks

Although dermal fillers are generally considered safe, several potential complications may arise. Filler migration, the displacement of filler material from its intended injection site, can result in unintended aesthetic outcomes. Encapsulation and nodule formation may occur if the body forms a fibrous capsule around the filler material, leading to palpable or visible nodules. Uneven distribution or incorrect injection techniques may also cause lumpiness and surface irregularities [68,69].

While such complications are relatively uncommon, occurring in fewer than 5% of procedures when performed by experienced practitioners, clinicians must maintain a comprehensive understanding of facial anatomy and filler characteristics to minimize these risks. It is essential to select appropriate fillers and employ precise injection techniques tailored to the patient’s unique facial anatomy and desired aesthetic outcomes. Thorough patient education regarding possible complications and emphasizing the importance of receiving treatments from qualified, experienced professionals is crucial for optimal results and patient safety [21,70].

### 3.2. Collagen Stimulators (PDO Powder)

In this review, the term ‘PDO powder’ is used generically to describe injectable collagen-stimulating formulations composed of micronized polydioxanone particles that are currently available in clinical practice, rather than a single proprietary product.

#### 3.2.1. Composition and Mode of Action

PDO powder is a biodegradable synthetic polymer extensively utilized in aesthetic medicine for its effective collagen-stimulating properties. Upon subdermal injection, PDO powder initiates a controlled inflammatory cascade, recruiting and activating fibroblasts, which subsequently synthesize collagen and elastin. This bioactive response enhances angiogenesis and promotes substantial remodeling of the extracellular matrix, thereby significantly improving structural integrity, skin elasticity, and overall dermal quality. In vitro investigations have demonstrated that PDO powder markedly elevates collagen production in cultured human fibroblasts, exhibiting significant increases within 24 to 48 h post-application. Comparative studies have also highlighted that PDO powder stimulates collagen synthesis more effectively than PLLA, suggesting its potential superiority as a collagen-inducing agent in aesthetic treatments [71,72,73].

Following subdermal administration, PDO powder undergoes predictable hydrolytic degradation, typically completed over several months. This gradual biodegradation process aligns synergistically with the body’s natural timeline for collagen remodeling, thus ensuring sustained tissue regeneration and continuous volumetric enhancement. The progressive degradation of PDO allows newly synthesized collagen fibers to effectively integrate into the dermal matrix, providing durable structural support and sustained aesthetic improvement. Published clinical evaluations confirm that PDO-induced collagen formation results in considerable enhancements in skin thickness, elasticity, hydration, and texture attributes essential for effectively addressing facial asymmetry with natural-looking outcomes [74,75,76,77].

Moreover, PDO powder has increasingly gained preference over PLLA due to several distinct physicochemical advantages. Firstly, PDO exhibits a more rapid biodegradation and absorption rate within human tissues compared to PLLA, significantly reducing the incidence of foreign body sensations and long-term tissue reactions. In contrast, PLLA’s slower biodegradation profile allows it to persist within tissues longer, potentially elevating the risk of prolonged foreign body sensations and associated complications [73]. Secondly, PDO demonstrates lower crystallinity relative to PLLA, substantially minimizing the risk of adverse events such as nodule formation or granuloma development within dermal layers. The reduced crystallinity contributes markedly to PDO’s favorable clinical safety profile, thereby decreasing the likelihood of adverse reactions and enhancing patient satisfaction [75]. Thirdly, PDO possesses a lower glass transition temperature than PLLA, making it inherently softer and more pliable under physiological conditions. This material flexibility ensures that PDO maintains a natural, rubber-like consistency within dermal tissues, enhancing patient comfort by providing a more natural tactile quality. Conversely, the higher glass transition temperature of PLLA results in a comparatively rigid and rough texture, potentially leading to palpable firmness and unnatural sensations beneath the skin [78]. Collectively, these distinct physicochemical attributes underscore PDO powder’s clinical superiority, highlighting its enhanced biocompatibility, improved patient comfort, and overall safety relative to PLLA.

#### 3.2.2. Clinical Applications and Effectiveness in Asymmetry Correction

PDO powder has shown significant efficacy in addressing facial asymmetry associated with volume deficits, skin laxity, and soft tissue irregularities. Its clinical applications encompass augmentation and sculpting of the cheeks, enhancement of the jawline, correction of nasolabial folds, and improvement of marionette lines. Empirical studies have demonstrated that PDO-based interventions lead to measurable enhancements in facial symmetry, more defined facial contours, and improved skin texture. Clinical evidence for injectable PDO powder in facial contouring and asymmetry correction is still emerging, but several observational studies and case series have reported improvements in physician-rated symmetry and patient satisfaction scores following treatment of the midface, jawline, and lower face [79]. In these reports, PDO powder is typically used to enhance soft-tissue volume and stimulate neocollagenesis in areas of relative deficiency, thereby complementing volumetric fillers and thread lifting procedures in multimodal treatment plans.

Following administration, PDO powder initiates collagen synthesis, with clinically noticeable outcomes typically emerging within 4 to 6 weeks post-treatment, aligning with the natural timeline of collagen production and extracellular matrix regeneration. These improvements, including enhanced skin thickness, elasticity, hydration, and texture, are crucial for effectively and naturally addressing facial asymmetry. The results generally persist for approximately 12 to 24 months, providing sustained and subtle aesthetic enhancements. This gradual collagen stimulation ensures that the outcomes not only appear natural but also continue to improve over time, making PDO powder a preferred option for patients seeking enduring corrections of facial asymmetry [73,74,80].

#### 3.2.3. Safety Profile and Patient Acceptance

The safety profile of PDO powder treatments is highly favorable. Common transient side effects include mild swelling, temporary bruising, erythema, and localized discomfort, which typically resolve spontaneously within days to a few weeks. Serious adverse events, such as granuloma formation or infections, are extremely rare, occurring in fewer than 1% of cases, particularly when injections are performed by experienced clinicians adhering strictly to aseptic protocols and precise injection techniques. The minimally invasive nature of PDO powder injections, combined with the material’s biocompatibility and predictable biodegradation profile, significantly contributes to its excellent safety record, making it a reliable and safe choice for facial rejuvenation and asymmetry correction [69,81].

Patient acceptance and satisfaction with PDO powder treatments are notably high, primarily attributable to their minimally invasive nature, gradual yet significant aesthetic improvements, and minimal downtime. Published clinical evaluations have reported patient satisfaction rates exceeding 85%, reflecting a strong appreciation for the subtle, progressive enhancement in facial symmetry, contour definition, and overall skin quality observed during the months following treatment. Furthermore, patients particularly value the ability to resume normal daily activities promptly, typically within 24 to 48 h post-procedure, further enhancing the appeal of PDO powder treatments as a practical and efficient option for long-lasting, natural-looking facial rejuvenation and asymmetry correction [82,83].

### 3.3. PDO Thread Lifting

#### 3.3.1. Technique and Mechanistic Basis

PDO thread lifting is an advanced, minimally invasive aesthetic technique involving the precise insertion of biodegradable PDO threads into subdermal tissues. These threads are strategically placed along predetermined anatomical vectors to mechanically lift, reposition, and stabilize sagging facial tissues, immediately enhancing facial contours and addressing skin laxity. PDO threads typically feature barbs or cones designed to securely anchor within subcutaneous tissues, ensuring immediate structural support and tissue repositioning. Additionally, the insertion of PDO threads initiates a biological response characterized by fibroblast activation and subsequent collagen synthesis. This neocollagenesis process progressively improves skin firmness, elasticity, and texture, resulting in sustained aesthetic benefits even after the threads are completely metabolized via hydrolysis over several months. This dual mechanism of immediate mechanical lifting combined with prolonged biological enhancement makes PDO thread lifting particularly effective for comprehensive and durable correction of facial asymmetry and contour irregularities [78,84,85,86].

In addition to providing immediate mechanical support, PDO threads stimulate biological tissue regeneration through activation of fibroblasts. This fibroblast stimulation initiates a cascade of new collagen, elastin, and HA synthesis within the dermal and subdermal layers, contributing significantly to sustained improvements in skin quality and volumetric restoration. The presence of PDO threads induces a controlled, localized inflammatory response, which further enhances extracellular matrix remodeling, reinforcing structural integrity, elasticity, and overall dermal resilience. Typically, PDO threads biodegrade completely through hydrolysis within 6–9 months post-treatment, yet the collagen and elastin deposition persists, resulting in prolonged aesthetic benefits. Consequently, this dual mechanical and biological mechanism facilitates lasting improvements in facial symmetry, skin firmness, texture, and elasticity, making PDO thread lifting an effective, long-lasting solution for addressing facial asymmetry and contour irregularities [87,88].

#### 3.3.2. Clinical Evidence and Patient Satisfaction

Published clinical evidence robustly supports the efficacy of PDO thread lifting in addressing facial asymmetry, skin laxity, and contour irregularities. Multiple clinical trials and observational studies have documented statistically significant improvements in facial symmetry, with measurable correction of midface ptosis, jawline definition, and overall aesthetic enhancement. Patient-reported outcomes consistently demonstrate high satisfaction rates, typically exceeding 80%, reflecting substantial improvements in both immediate facial contouring and progressive, collagen-mediated skin rejuvenation. The dual benefit of instantly noticeable lifting effects combined with ongoing neocollagenesis ensures sustained improvement in facial aesthetics, further increasing patient acceptance and adherence to treatment recommendations. Collectively, these advantages position PDO thread lifting as a valuable and highly effective non-surgical modality for the durable correction of facial asymmetry and enhancement of overall facial appearance [82,83,85].

The aesthetic outcomes of PDO thread lifting typically persist for approximately 12 to 18 months, corresponding closely with the threads’ biodegradation timeline. Published clinical follow-up studies consistently report high patient satisfaction, often exceeding 85%, reflecting the procedure’s effective combination of immediate visible improvement and subsequent progressive collagen remodeling. Factors contributing to elevated patient acceptance include the minimally invasive nature of the procedure, reduced procedural discomfort, limited recovery time typically ranging from just a few days to one week and gradual, natural-looking enhancement in facial symmetry and skin quality. These attributes make PDO thread lifting particularly appealing to patients seeking a safe, effective, and non-surgical alternative to conventional facelift surgeries, offering both immediate aesthetic gratification and sustained, long-term rejuvenation [85,89].

#### 3.3.3. Specific Considerations in Facial Palsy-Related Asymmetry

**Figure 2:**
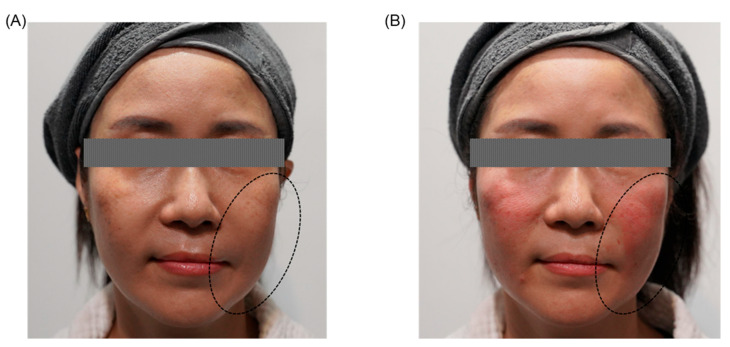
Representative clinical case from an aesthetic clinic in Korea of a female patient in her fifties presenting significant facial asymmetry due to left-sided facial nerve palsy, characterized by decreased muscular strength and pronounced skin laxity affecting the entire left side of the face. (**A**) Before procedure. (**B**) After a single session of PDO thread lifting applied to the whole face. The circled areas on the patient photographs indicate the specific treatment sites, illustrating immediate and substantial improvements in facial symmetry, muscle tone, skin tightening, and overall aesthetic balance following the procedure.

PDO thread lifting is particularly beneficial for addressing facial asymmetry associated with facial palsy, a condition characterized by muscular weakness, atrophy, and soft tissue ptosis, significantly impacting facial symmetry, function, and patient quality of life. PDO threads facilitate targeted mechanical repositioning and elevation of affected soft tissues, providing immediate and meaningful restoration of facial balance (Figure 2). Due to the compromised muscular tone and altered facial biomechanics commonly seen in facial palsy patients, meticulous planning and precise thread placement are crucial. Customized insertion vectors must account for the unique anatomical challenges and weakened musculature, ensuring optimal restoration of both aesthetic harmony and functional outcomes, such as improved oral competence and eyelid closure. Consequently, PDO thread lifting represents an effective, minimally invasive approach for substantially enhancing the quality of life and self-esteem of patients experiencing facial palsy-related asymmetry [24]. In patients with long-standing facial nerve palsy, gravitational descent and muscular imbalance frequently result in marked facial asymmetry, particularly in the midface, perioral, and lower face regions. PDO thread lifting can be used to reposition ptotic tissues, support weakened soft-tissue structures, and restore more balanced facial contours on the affected side. Published clinical reports have demonstrated that carefully planned vector-based PDO thread placement improves static symmetry, enhances patient-perceived attractiveness, and may partially compensate for dynamic asymmetry by providing mechanical support to the paralyzed side [90,91]. In our clinical practice, PDO threads are often combined with volumizing fillers or collagen stimulators to address both vertical descent and volumetric deficiencies in facial palsy patients.

Effective correction of facial palsy-related asymmetry using PDO threads necessitates meticulous pre-procedural planning and precise thread placement, carefully tailored to each patient’s unique anatomical alterations and impaired muscular dynamics. Clinicians must strategically determine optimal insertion vectors to maximize mechanical repositioning effects, adequately accommodating weakened muscles, connective tissue laxity, and altered facial biomechanics characteristic of facial palsy. Given the complexity and variability of anatomical and functional changes associated with facial palsy, a comprehensive understanding of facial anatomy, neuromuscular interactions, and the specific biomechanical alterations present is essential. This advanced knowledge enables clinicians to achieve balanced, natural, and functionally beneficial outcomes while significantly reducing the risk of complications such as inadequate tissue repositioning, visible irregularities, or diminished procedural efficacy. Consequently, specialized expertise and careful consideration of these factors significantly enhance both functional and aesthetic results, improving patient quality of life and satisfaction [24,92].

### 3.4. Energy-Based Non-Invasive Devices

#### 3.4.1. Types of Devices and Mechanisms of Collagen Remodeling

Energy-based non-invasive devices represent a diverse group of aesthetic technologies, prominently including radiofrequency (RF), ultrasound, and laser therapies. These modalities share a common objective: inducing controlled thermal or mechanical injury to the skin’s deeper layers to stimulate collagen remodeling and neocollagenesis [22,93,94].

RF devices employ electromagnetic energy to generate controlled thermal energy within dermal and subdermal tissues, typically achieving penetration depths ranging from 1.5 to 4.0 mm. Upon application, RF-induced heating (optimally maintained between approximately 55 °C and 65 °C) initiates immediate contraction and denaturation of collagen fibers, subsequently triggering fibroblast activation and sustained collagen and elastin synthesis. This dual-phase response an initial tightening effect followed by progressive tissue remodeling results in clinically appreciable improvements in skin firmness, elasticity, texture, and overall structural integrity. RF devices vary in their design, encompassing monopolar, bipolar, and multipolar systems, each characterized by distinct thermal delivery mechanisms and targeted tissue depths. Monopolar systems achieve deeper tissue penetration, making them suitable for substantial volume tightening and contouring, whereas bipolar and multipolar systems deliver energy superficially, effectively addressing skin laxity, fine lines, and texture irregularities. Consequently, the selection of an RF modality must be precisely tailored to individual patient needs, anatomical considerations, and specific aesthetic objectives to optimize outcomes and ensure procedural safety [95,96].

Ultrasound-based devices, particularly high-intensity focused ultrasound (HIFU), employ high-frequency acoustic energy that is precisely delivered to targeted tissue depths ranging from approximately 1.5 to 4.5 mm below the epidermal surface. At these specified depths, the focused ultrasound energy generates discrete and controlled zones of thermal coagulation (approximately 60–70 °C), selectively triggering a pronounced regenerative response through immediate collagen denaturation followed by sustained neocollagenesis and elastin synthesis. Crucially, the precise focal nature of ultrasound energy delivery ensures preservation of the epidermal integrity, significantly minimizing the risk of superficial tissue damage and downtime. Over subsequent weeks to months, this robust tissue remodeling process substantially enhances dermal density, elasticity, and skin firmness, leading to visibly improved facial contours. Due to its ability to penetrate deeply and accurately, ultrasound therapy is particularly advantageous for managing moderate to severe skin laxity, structural asymmetries, and contour irregularities, providing enduring aesthetic outcomes with a high patient satisfaction profile [97,98].

Laser therapies comprise a diverse array of technologies, notably including ablative fractional CO_2_ lasers and non-ablative fractional lasers, each employing specific wavelengths of coherent light energy to achieve distinct clinical outcomes tailored to aesthetic goals and tissue characteristics. Ablative fractional CO_2_ lasers operate by precisely generating microscopic columns of thermal injury (approximately 100–300 µm in diameter) that penetrate deeply into the epidermal and upper dermal layers. This controlled epidermal ablation initiates an intense regenerative response, characterized by significant fibroblast activation, robust neocollagenesis, and elastin synthesis, culminating in substantial dermal remodeling and skin rejuvenation. Conversely, non-ablative fractional lasers induce subepidermal thermal coagulation without removing superficial epidermal layers, thereby preserving epidermal integrity, minimizing downtime, and enhancing patient comfort. Both modalities effectively stimulate dermal remodeling processes, substantially improving skin texture, firmness, and elasticity, reducing fine lines, wrinkles, and photodamage, and correcting mild to moderate facial asymmetries. Selection between ablative and non-ablative lasers depends on individual patient characteristics, severity of skin laxity, desired recovery times, and treatment objectives, allowing clinicians to offer highly customized and efficacious rejuvenation strategies [99,100].

#### 3.4.2. Clinical Efficacy, Limitations, and Recommended Treatment Protocols

**Figure 3:**
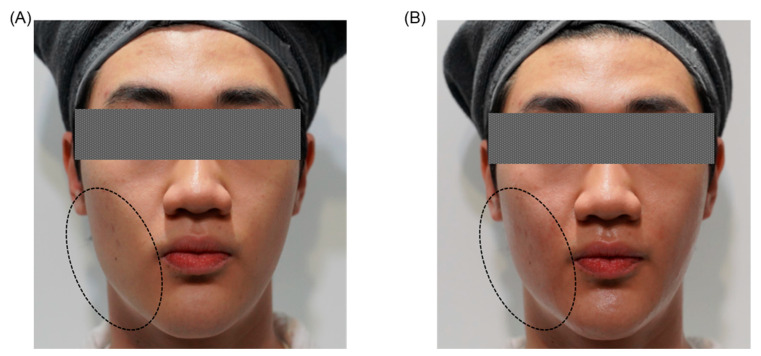
Representative clinical case of an adolescent male patient treated at an aesthetic clinic in Korea, presenting mild facial asymmetry characterized by muscle hypertrophy and lower facial fullness predominantly on the right side. (**A**) Before procedure. (**B**) After a single session of non-invasive radiofrequency (RF) lifting treatment. The circled areas on the patient photographs indicate the specific treatment sites, clearly illustrating immediate improvement in facial symmetry, reduced muscular prominence, enhanced contour definition, and overall aesthetic balance following the procedure.

Published clinical studies consistently validate the efficacy of energy-based non-invasive devices including radiofrequency, ultrasound (HIFU), and laser therapies in addressing facial asymmetry, enhancing skin laxity, and significantly refining skin texture and elasticity (Figure 3). Empirical evidence demonstrates notable and measurable improvements in facial contours, symmetry, and overall skin quality, driven primarily by a robust and sustained collagen remodeling process initiated by targeted dermal heating. Clinical outcomes typically become apparent within several weeks, with continued enhancements occurring progressively over 3 to 6 months following treatment, correlating closely with the body’s natural timeline for collagen synthesis and extracellular matrix reorganization. The longevity of these improvements varies considerably based on device type, specific treatment parameters, anatomical region, and individual patient characteristics, with clinical durability typically ranging from approximately 6 months to as long as 2 years. This variation underscores the importance of tailored treatment protocols and periodic maintenance sessions, optimized according to patient-specific anatomical considerations and desired aesthetic outcomes [101,102].

Despite the demonstrated clinical efficacy of energy-based non-invasive treatments, several notable limitations warrant consideration. Patient responses exhibit considerable variability, influenced significantly by individual factors such as chronological age, skin phototype, baseline collagen density, and the severity of existing tissue laxity. Specifically, older patients or those with advanced dermal elastosis often exhibit attenuated responses, necessitating careful patient selection and realistic expectation management. Furthermore, optimal aesthetic outcomes typically require multiple treatment sessions commonly ranging from three to six sessions spaced at regular intervals of approximately 4–6 weeks. This treatment schedule, though essential for maximal collagen remodeling and sustained tissue improvement, can potentially challenge patient adherence, impact overall satisfaction, and necessitate comprehensive patient education and counseling to ensure compliance and enhance long-term treatment success [103,104,105].

Recommended treatment protocols for energy-based non-invasive devices generally consist of an initial series of multiple sessions typically three to six treatments administered at intervals of approximately four to six weeks, tailored according to the specific device employed and targeted clinical indications. Following completion of the initial treatment regimen, periodic maintenance sessions, typically scheduled every 6 to 12 months, are recommended to sustain and prolong the achieved aesthetic improvements. Clinicians must meticulously select treatment parameters, factoring in individual patient characteristics, including skin phototype, baseline collagen density, severity of tissue laxity, and patient-specific aesthetic goals. Comprehensive patient education and counseling regarding realistic expectations, potential outcomes, anticipated improvement timelines, and the importance of adhering to recommended treatment schedules are essential for optimizing patient satisfaction, promoting compliance, and ensuring long-term treatment success [106,107].

### 3.5. Extracorporeal Shockwave Therapy

#### 3.5.1. Overview and Types

ESWT is an advanced, non-invasive therapeutic modality that utilizes high-energy acoustic waves to induce targeted biological responses within treated tissues. These acoustic waves are characterized by rapid pressure fluctuations, substantial peak energy, and precise waveform delivery, enabling effective propagation through biological structures and controlled stimulation of tissue regeneration processes. ESWT is classified into two main modalities: radial shockwave therapy (RSWT) and focused shockwave therapy (FSWT). Radial shockwaves disperse acoustic energy broadly, predominantly affecting superficial tissues, and are effective for generalized treatment of larger areas. In contrast, focused shockwaves concentrate energy at specific focal points deeper within tissue layers, providing precise, high-intensity therapeutic stimulation suitable for targeted and localized treatment areas. Understanding these distinctive mechanisms and selecting the appropriate modality based on treatment depth and specific therapeutic goals is essential for achieving optimal clinical outcomes in addressing facial asymmetry, tissue laxity, and soft-tissue rejuvenation [108,109].

Radial shockwave therapy (RSWT) utilizes acoustic waves that propagate radially from the applicator head, broadly dispersing energy across superficial tissue structures, typically reaching depths of approximately 1 to 3 cm. Due to its diffuse energy distribution, RSWT is particularly effective in treating conditions involving superficial muscle groups, connective tissues, and fascia near the skin surface, offering notable benefits for general soft-tissue rejuvenation and mild-to-moderate tissue laxity. Conversely, focused shockwave therapy (FSWT) delivers highly concentrated acoustic waves precisely targeted to deeper tissue layers, typically at depths ranging from 3 to 6 cm. This focused delivery enables a more intense and localized therapeutic response, effectively triggering robust cellular regeneration, enhanced fibroblast-mediated collagen synthesis, and significant improvements in tissue remodeling and healing processes. Consequently, FSWT is particularly advantageous for addressing deeper structural deficits and substantial facial asymmetry, providing pronounced and sustained improvements in tissue integrity, elasticity, and overall aesthetic outcomes. The strategic selection between radial and focused shockwave modalities, guided by specific anatomical considerations and treatment objectives, is essential for optimizing therapeutic efficacy [108,110,111].

#### 3.5.2. Mechanism

The therapeutic efficacy of ESWT is primarily attributed to its dual mechanism involving mechanical stimulation and subsequent biological tissue regeneration. Upon application, acoustic shockwaves induce precisely controlled microtrauma within targeted tissues, triggering a localized and transient inflammatory cascade characterized by enhanced cytokine and growth factor release. This carefully regulated inflammatory environment accelerates cellular proliferation and fibroblast activation, leading to robust collagen synthesis, elastin production, and extracellular matrix remodeling. Additionally, ESWT markedly promotes angiogenesis, significantly enhancing local microvascular density and blood perfusion to treated tissues. This increased vascularity not only expedites the regenerative processes but also improves oxygenation and nutrient delivery, further supporting tissue healing, structural integrity, and overall aesthetic improvements. Consequently, ESWT offers substantial therapeutic benefits for addressing facial asymmetry, skin laxity, and various soft-tissue deficiencies through combined mechanical stimulation and enhanced biological regeneration [111,112,113].

Additionally, ESWT significantly enhances neocollagenesis through targeted fibroblast activation, markedly increasing the synthesis of collagen, elastin, and other critical extracellular matrix components. This intensified matrix remodeling substantially contributes to enhanced structural integrity, elasticity, and overall rejuvenation of treated skin and underlying connective tissues. Furthermore, ESWT exerts a direct stimulatory effect on muscular tissues, optimizing muscular tension regulation and improving muscle fiber function, which collectively supports tissue elasticity and functional recovery. The acoustic waves generated by ESWT induce elevated local metabolic activity and significantly augment blood circulation, accelerating nutrient supply and removal of metabolic byproducts. This comprehensive therapeutic response effectively expedites tissue repair processes, delivering meaningful improvements in both structural rejuvenation and functional recovery outcomes. Consequently, ESWT serves as a potent modality for addressing facial asymmetry, improving tissue laxity, and promoting comprehensive dermal and subdermal tissue rejuvenation [114,115].

#### 3.5.3. Clinical Outcomes and Cumulative Effects

Published clinical studies consistently validate the efficacy of ESWT for effectively addressing facial asymmetry, enhancing skin texture, and significantly improving overall soft-tissue quality. Empirical evidence indicates that optimal aesthetic and structural outcomes typically require a cumulative approach involving multiple treatment sessions usually ranging from three to six sessions administered at intervals of approximately one to two weeks. Noticeable improvements in facial contours, symmetry, and skin quality commonly become evident progressively throughout the treatment course, with peak results typically manifesting several weeks to months following therapy completion. These sustained enhancements are primarily attributable to ongoing, robust neocollagenesis, extracellular matrix remodeling, and increased tissue vascularization induced by repeated acoustic stimulation. Consequently, ESWT is recognized as a highly effective non-invasive therapeutic strategy for achieving progressive, natural, and long-lasting correction of facial asymmetry and comprehensive skin rejuvenation [116,117,118].

The therapeutic effects of ESWT are typically sustained over several months, with periodic maintenance sessions often recommended every 4 to 6 months advised to prolong and enhance aesthetic outcomes. Clinical evaluations frequently report high patient satisfaction rates, often exceeding 85%, attributable to the treatment’s non-invasive characteristics, favorable safety profile, and minimal side effects, such as transient erythema or mild discomfort. Patients particularly value ESWT’s ability to deliver gradual yet significant improvements in facial symmetry, contour refinement, and overall skin quality without significant disruption to daily activities, typically resuming routine tasks immediately following each treatment session. The combination of progressive, natural-looking aesthetic enhancements and convenience positions ESWT as a highly appealing and patient-preferred modality for long-term facial rejuvenation and effective correction of facial asymmetry [116,119]. A comparative summary of these non-surgical modalities including their mechanisms, duration, advantages, and limitations is presented in Table 3.

## 4. Advantages of Non-Surgical Facial Asymmetry Correction

### 4.1. Convenience of Procedure and Immediate Return to Daily Activities

Non-surgical approaches for correcting facial asymmetry offer significant advantages in terms of procedural convenience and rapid recovery compared to conventional surgical interventions. Minimally invasive modalities, such as dermal fillers, PDO thread lifting, energy-based devices (e.g., RF, ultrasound, laser therapies), and ESWT, are efficiently conducted as outpatient procedures, typically completed within approximately 30–60 min. This streamlined treatment process eliminates the need for inpatient admission, extensive anesthesia, and prolonged medical monitoring. Consequently, patients benefit from minimal disruption to their schedules, swiftly resuming routine professional and social activities. This procedural convenience, combined with reduced recovery periods and negligible downtime, substantially enhances patient satisfaction and compliance, making non-surgical methods a highly attractive and practical solution for effective management of facial asymmetry and rejuvenation [24,120].

Due to the minimally invasive nature of these non-surgical procedures, tissue trauma is significantly reduced, and surgical incisions are virtually absent, resulting in minimal postoperative discomfort. Patients typically report only mild, transient side effects including slight swelling, minor bruising, or temporary erythema at the injection or treatment sites which usually resolve spontaneously within 1 to 3 days without the need for medical intervention. Consequently, associated downtime is exceptionally brief, allowing patients to promptly return to their normal professional, social, and recreational activities without significant disruption. This favorable recovery profile greatly contributes to patient convenience, acceptance, and overall satisfaction, positioning non-surgical facial asymmetry correction techniques as highly attractive alternatives to traditional surgery [121].

The rapid recovery and minimal downtime associated with non-surgical correction methods considerably enhance patient convenience and significantly elevate overall patient satisfaction. This favorable recovery profile is especially appealing to individuals with active lifestyles, professional commitments, and limited availability, who seek effective and noticeable aesthetic improvements without disrupting their daily routines and responsibilities. Consequently, the combination of immediate procedural convenience, minimal post-treatment limitations, and the ability to quickly return to normal activities positions non-surgical facial asymmetry correction as an optimal choice for patients prioritizing both efficacy and minimal interference with their lifestyle demands [122,123].

### 4.2. Minimal Invasiveness and Reduced Discomfort

Non-surgical treatments for facial asymmetry offer distinct advantages due to their minimally invasive nature and significantly enhanced patient comfort compared to traditional surgical approaches. These methods commonly utilize topical anesthetics or precise local anesthetic injections, effectively minimizing procedural discomfort, reducing patient anxiety, and facilitating a relaxed and comfortable treatment experience. In contrast to surgical interventions which typically involve extensive tissue dissection, incisions, substantial manipulation of anatomical structures, and prolonged recovery periods often accompanied by considerable postoperative pain non-surgical techniques markedly diminish these challenges. Consequently, patients undergoing non-surgical interventions experience fewer adverse effects, reduced discomfort during and after the procedure, and significantly accelerated recovery, collectively enhancing patient acceptance, satisfaction, and overall treatment adherence [20,124,125].

Patients frequently report minimal discomfort during and following non-surgical facial asymmetry correction procedures, typically describing sensations as mild, brief, and easily manageable. The negligible pain associated with these minimally invasive interventions substantially enhances patient comfort, contributing directly to greater patient acceptance, higher satisfaction rates, and improved treatment adherence. This favorable pain profile positions non-surgical methods as an attractive, practical, and highly preferable alternative for patients seeking effective facial asymmetry correction while intentionally avoiding the invasiveness, prolonged recovery, and considerable discomfort typically associated with conventional surgical procedures [126]. Despite the excellent safety profile of non-surgical treatments, occasional adverse events have been reported. These complications and their recommended management strategies are summarized in Table 4.

### 4.3. Rapid and Progressive Aesthetic Results

Non-surgical facial asymmetry correction techniques, notably PDO powder and PDO thread lifting, offer immediate visible improvements, providing patients with immediate aesthetic enhancements directly following treatment. Beyond this immediate effect, these methods initiate a robust biological response characterized by sustained collagen regeneration, typically commencing approximately four weeks post-procedure. The progressive collagen synthesis continually refines and enhances the initial corrections, fostering ongoing improvements in facial symmetry, skin elasticity, texture, and overall dermal integrity. Consequently, patients benefit from dual-phase aesthetic outcomes a prompt initial correction complemented by continuous, natural-looking improvement, resulting in long-lasting and progressively enhanced facial rejuvenation [73,80].

**Figure 4:**
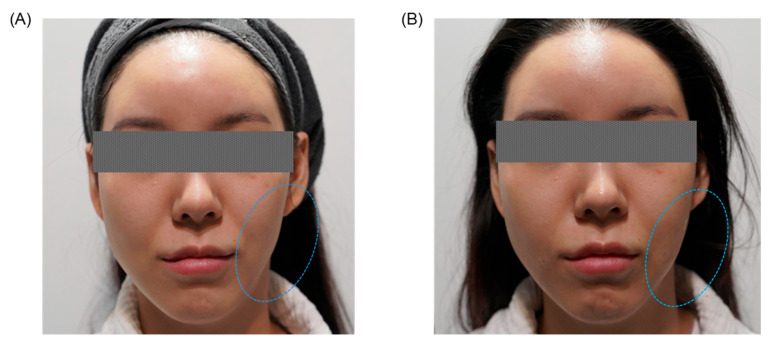
Representative clinical case of a female patient in her twenties treated at an aesthetic clinic in Korea, presenting significant facial asymmetry, primarily characterized by volume deficiency on the left side of the face. (**A**) Before procedure. (**B**) After a single session of non-invasive radiofrequency (RF) lifting combined with a collagen stimulator (PDO powder) administered via cannula. The circled areas in the patient photographs indicate the specific treatment sites, clearly illustrating substantial improvement in facial symmetry, volumetric restoration, and overall aesthetic balance following the combined procedure.

The combined advantage of immediate visual results and progressive, cumulative aesthetic enhancements significantly elevates patient satisfaction with non-surgical facial asymmetry correction methods (Figure 4). Patients frequently report exceptionally high satisfaction rates, driven by the immediate and noticeable improvements in facial contour and symmetry, complemented by the ongoing, natural evolution of outcomes. This gradual yet consistent improvement, facilitated by sustained collagen remodeling and tissue regeneration, ensures balanced, harmonious, and enduring aesthetic results. Ultimately, these procedures effectively meet patients’ expectations by providing both immediate aesthetic gratification and long-lasting, comprehensive facial rejuvenation [86,126].

### 4.4. Versatility and Effective Integration into Clinical Practice

Non-surgical methods for correcting facial asymmetry exhibit substantial versatility, effectively addressing diverse clinical objectives including facial rejuvenation, soft tissue lifting, volume restoration, and contour enhancement. The inherent adaptability of these minimally invasive techniques allows clinicians to meticulously tailor treatment protocols according to individual anatomical features, specific aesthetic concerns, and personalized patient expectations. Such precise customization facilitates optimal clinical outcomes by aligning treatment strategies closely with each patient’s unique goals and anatomical characteristics. Consequently, this individualized approach not only enhances procedural efficacy but also ensures precise fulfillment of therapeutic objectives, consistently achieving natural, harmonious, and highly satisfactory aesthetic results [20,127].

Patients who previously viewed surgical interventions as their sole viable treatment option frequently express high levels of satisfaction upon discovering minimally invasive alternatives for facial asymmetry correction. This increased satisfaction arises from the markedly reduced invasiveness, significantly lower risk profile, rapid recovery, and highly customized aesthetic outcomes achievable through non-surgical approaches. Incorporating these versatile, minimally invasive modalities into clinical practice significantly broadens the therapeutic repertoire, allowing clinicians to effectively address a wider spectrum of aesthetic concerns. Consequently, this approach enhances patient satisfaction and retention rates, attracts a broader patient demographic, and enriches clinical practice by accommodating diverse patient needs, treatment expectations, and aesthetic objectives with personalized, safe, and efficacious outcomes [128]. From a practical standpoint, cost is a critical factor influencing the choice between surgical and non-surgical correction. In general, non-surgical approaches entail lower upfront costs and markedly shorter downtime than orthognathic surgery, enabling many patients to continue their daily activities and work with minimal interruption. However, the need for repeated maintenance sessions and the variability of product and device pricing across different healthcare systems must be considered. These cost-related issues should be discussed transparently with patients as part of shared decision-making when planning individualized treatment strategies for facial asymmetry.

## 5. Discussion and Limitations

### 5.1. Discussion

Non-surgical facial asymmetry correction offers highly effective and minimally invasive alternatives to traditional surgical approaches, substantially enhancing patient comfort, reducing recovery time, and significantly increasing overall satisfaction. Advanced techniques, including dermal fillers, PDO thread lifting, energy-based modalities, and ESWT, provide immediate visible improvements along with progressive aesthetic enhancements driven by sustained collagen regeneration, thus delivering balanced, harmonious, and enduring results. Clinicians may use the etiologic classification proposed in this review volume-related, soft-tissue sagging, dynamic, superficial, and composite asymmetry as a practical framework when selecting combinations of dermal fillers, collagen stimulators, thread lifting, and energy-based devices. By matching the dominant etiologic pattern to the mechanism of action of each modality, individualized, stepwise treatment plans can be constructed for patients who present with diverse forms of facial asymmetry in everyday practice. Future research should focus on well-designed comparative trials, standardized and quantitative measures of facial symmetry, long-term safety and durability data for non-surgical modalities, and robust cost-effectiveness analyses compared with surgical correction. Addressing these gaps will refine clinical decision-making and further optimize non-surgical management of facial asymmetry.

### 5.2. Limitations

This narrative review has several limitations. First, we did not apply a formal, tool-based risk-of-bias assessment, and the available evidence is dominated by small, single-center observational studies and case series. These designs are inherently prone to selection, reporting, and publication bias, which may overestimate treatment effectiveness and underreport adverse events. Second, the heterogeneity of study designs, outcome measures, and follow-up durations precluded any quantitative synthesis or direct comparison between modalities. Therefore, the conclusions drawn from the current literature should be interpreted with caution, and high-quality comparative clinical trials are needed to strengthen the evidence base.

## Figures and Tables

**Table 1:** Clinical categories of facial asymmetry and their key clinical findings.

Clinical Category	Key Clinical Findings
**Volume-related Asymmetry**	Uneven cheek contours, lip asymmetry, infraorbital hollowing (tear trough deformity)
**Soft Tissue Sagging and Skin Laxity**	Drooping eyebrows, accentuated nasolabial folds, irregular jawlines (jowls formation)
**Dynamic (Muscular) Asymmetry**	Asymmetric facial expressions, uneven smiles, involuntary muscle contractions (synkinesis)
**Superficial Skin Texture Irregularities**	Fine lines, wrinkles, acne scars, uneven skin texture and tone
**Composite (Mixed) Asymmetry**	A combination of volume depletion, skin laxity, muscular imbalance, and superficial skin irregularities

**Table 2:** Non-surgical aesthetic modalities and their key mechanisms and clinical effects.

Aesthetic Modality	Key Mechanisms and Clinical Effects
**Dermal Fillers**	Immediate volume restoration; stimulates collagen production, enhances facial symmetry and contours
**Collagen Stimulators (Polydioxanone Powder)**	Gradual biostimulation; significant collagen synthesis, enhances skin elasticity, volume, and structural support
**Polydioxanone Thread Lifting**	Immediate mechanical lifting; stimulates collagen synthesis, provides structural support, enhances skin elasticity and facial contours
**Energy-based Non-invasive Devices**	Stimulates dermal collagen remodeling through controlled thermal/mechanical injury; improves skin firmness, elasticity, and contours
**Extracorporeal Shockwave Therapy**	Mechanical stimulation inducing biological tissue regeneration; promotes neocollagenesis, angiogenesis, improves tissue elasticity and symmetry

**Table 3:** Comparative summary of major non-surgical modalities used for facial asymmetry correction, highlighting their primary mechanisms, effect duration, advantages and potential limitations from recent clinical literature.

Modality	Mechanism of Action	Onset of Effect	Duration of Effect	Major Advantages	Limitations/Complications
**Dermal Fillers (HA, CaHA, PLLA, PMMA)**	Immediate volume restoration and collagen stimulation	Immediate to 1 week	6–24 months (depends on filler type)	Instant results, reversible (HA), customizable	Edema, bruising, nodule or migration (<5%)
**Collagen Stimulator (PDO Powder)**	Induces fibroblast activation and neocollagenesis	4–6 weeks	12–24 months	Gradual natural correction, safe biodegradation, minimal downtime	Mild swelling, erythema, rare granuloma (<1%)
**PDO Thread Lifting**	Mechanical lifting + collagen induction	Immediate	12–18 months	Dual effect (instant lift + collagen), minimal invasiveness	Temporary dimpling, mild asymmetry, thread migration (<5%)
**Energy-based Devices (RF, HIFU, Laser)**	Controlled dermal heating → collagen remodeling	2–4 weeks	6–24 months	Non-invasive, improves texture and firmness	Multiple sessions required, transient erythema
**Extracorporeal Shockwave Therapy (ESWT)**	Mechanical acoustic stimulation → angiogenesis and collagen synthesis	Gradual (2–4 weeks)	6–12 months	Enhances elasticity, no downtime, safe	Mild erythema or tenderness

**Table 4:** Reported complications and management strategies of non-surgical modalities for facial asymmetry correction.

Modality	Representative Complication	Estimated Incidence	Underlying Cause/Mechanism	Recommended Management or Prevention
**Dermal Fillers (HA, CaHA, PLLA, PMMA)**	Edema, bruising, nodule formation, vascular occlusion (rare)	<5%	Improper injection depth, intravascular injection, overcorrection	Gentle massage for minor irregularities; hyaluronidase for HA fillers; avoid high-pressure injection; use aspiration technique
**Collagen Stimulator (PDO Powder)**	Mild swelling, erythema, transient tenderness	<3%	Local inflammatory reaction to polymer degradation	Cold compress, short-course NSAIDs; adhere to aseptic injection technique
**PDO Thread Lifting**	Thread migration, surface irregularity, dimpling, asymmetry	3–5%	Inaccurate vector placement, superficial insertion, excessive tension	Early manual correction or thread removal; meticulous vector planning; proper depth and tension control
**Energy-based Devices (RF, HIFU, Laser)**	Transient erythema, edema, rare burns or dyschromia	1–3%	Excessive energy or overlap of treatment passes	Cooling, topical steroid or emollient; proper energy calibration and operator training
**Extracorporeal Shockwave Therapy (ESWT)**	Temporary erythema, tenderness, petechiae	<2%	Local mechanical stress or vascular dilation	Usually self-limited; avoid high energy density and maintain optimal probe contact

## Data Availability

Data is contained within the article.

## Abbreviations

PDO, polydioxanone; RF, radiofrequency; ESWT, extracorporeal shockwave therapy; HA, hyaluronic acid; CaHA, calcium hydroxylapatite; PLLA, poly-L-lactic acid; PMMA, polymethylmethacrylate; HIFU, high-intensity focused ultrasound.

## References

[1] Guennouni A., Aallah W.H., Abourak C., Oukassem S., Ech-Cherif El Kettani N., Fikri M., Touarsa F., Jiddane M., Zekri M., Bellakhdar M. (2025). A facial asymmetry revealed: Active mandibular condylar hyperplasia. Radiol. Case Rep..

[2] Shackelford T.K., Larsen R.J. (1997). Facial asymmetry as an indicator of psychological, emotional, and physiological distress. J. Pers. Soc. Psychol..

[3] Bakri M.M.H., Vishvnathaiah S., Bakmani H.F., Hakami A.J., Zaidan M.S., Dighriri M.A., Jad Y.A., Hakami T.M., Bakri H.M.H. (2024). Prevalence of mandibular asymmetries in the pediatric population of Jazan: A radiographic analytical study. Heliyon.

[4] Chojdak-Łukasiewicz J., Paradowski B. (2022). Facial Asymmetry: A Narrative Review of the Most Common Neurological Causes. Symmetry.

[5] Piao Y., Kim S.J., Yu H.S., Cha J.Y., Baik H.S. (2016). Five-year investigation of a large orthodontic patient population at a dental hospital in South Korea. Korean J. Orthod..

[6] Reddy N.V.V., Potturi A., Rajan R., Jhawar D., Bharath Bhushan Y.W., Pasupuleti A. (2023). Facial Asymmetry-Demystifying the Entity. J. Maxillofac. Oral. Surg..

[7] La Touche R., Losana-Ferrer A., Pascual-Vaquerizo E., Suso-Martí L., Paris-Alemany A., Chamorro-Sánchez J., Cuenca-Martínez F. (2019). Orofacial sensorimotor behaviour in unilateral chewing: A comparative analysis in asymptomatic population. Physiol. Behav..

[8] Ren L., Chen P., Musa M., Zhao Y., Awad R., Xiao Z., Li C., Li D., Chen X. (2025). Quantitative and qualitative condylar changes Post-Stabilization splint in patients with temporomandibular disorder and chewing side preference. Sci. Rep..

[9] Heikkinen E.V., Vuollo V., Heikkinen T., Harila V. (2024). Chewing Side Preference, Facial Asymmetry and Related Factors in the Northern Finland Birth Cohort 1986. Acta Odontol. Scand..

[10] Tran C., Choi D., Ko A.C., Carter K.D., Shriver E.M. (2022). The Effect of Sleep Position Preference on Eyelid and Eyebrow Symmetry. Ophthalmic Plast. Reconstr. Surg..

[11] Ko E.W., Huang C.S., Lin C.-H., Chen Y.-R. (2022). Orthodontic Perspective for Face Asymmetry Correction. Symmetry.

[12] Kim S.Y., Choi Y.H., Kim Y.K. (2018). Postoperative malocclusion after maxillofacial fracture management: A retrospective case study. Maxillofac. Plast. Reconstr. Surg..

[13] Chen G., Hsieh E.Y., Chen S.H., Pai B.C.J., Tsai C.Y., Wang S.W., Chou P.Y. (2023). Occlusion-Based Three-Dimensional Craniofacial Anthropometric and Symmetric Evaluation in Preadolescences: A Comparative COHORT Study. J. Clin. Med..

[14] Otel A., Montiel-Company J.M., Zubizarreta-Macho Á. (2025). Comparative Analysis of Early Class III Malocclusion Treatments—A Systematic Review and Meta-Analysis. Children.

[15] Inchingolo A.M., Patano A., Piras F., Ruvo E.D., Ferrante L., Noia A.D., Dongiovanni L., Palermo A., Inchingolo F., Inchingolo A.D. (2023). Orthognathic Surgery and Relapse: A Systematic Review. Bioengineering.

[16] Neeraj, Reddy S.G., Dixit A., Agarwal P., Chowdhry R., Chug A. (2021). Relapse and temporomandibular joint dysfunction (TMD) as postoperative complication in skeletal class III patients undergoing bimaxillary orthognathic surgery: A systematic review. J. Oral Biol. Craniofac. Res..

[17] Al-Moghrabi D., Salazar F.C., Pandis N., Fleming P.S. (2017). Compliance with removable orthodontic appliances and adjuncts: A systematic review and meta-analysis. Am. J. Orthod. Dentofac. Orthop..

[18] Stefanovic N.L., Uhac M., Brumini M., Zigante M., Perkovic V., Spalj S. (2021). Predictors of patient compliance during Class II division 1 malocclusion functional orthodontic treatment. Angle Orthod..

[19] Kim H.J., Noh H.K., Park H.S. (2023). Nonsurgical orthodontic correction of facial asymmetry by condylar remodeling and mandibular repositioning following occlusal cant correction with microimplants: A case report. Angle Orthod..

[20] Li K., Meng F., Li Y.R., Tian Y., Chen H., Jia Q., Cai H., Jiang H.B. (2022). Application of Nonsurgical Modalities in Improving Facial Aging. Int. J. Dent..

[21] Mahmood Faris B.J. (2024). The Use of Facial Fillers in Clinical Practice: The Level of Patient Satisfaction and an Overview of Common Clinical Complications. Actas Dermo-Sifiliogr..

[22] Jia X., Feng Y. (2025). Energy-Based Skin Rejuvenation: A Review of Mechanisms and Thermal Effects. J. Cosmet. Dermatol..

[23] Wigley C.H., Janssen T.J., Mosahebi A. (2023). Shock Wave Therapy in Plastic Surgery: A Review of the Current Indications. Aesthet. Surg. J..

[24] Bhatnagar A., Rai R., Kumar S., Mitra B., Chopra A., Singh G.K., Mitra D., Patil C., Sandhu S. (2022). Safety and Efficacy of Restoring Facial Symmetry Using Polydioxanone Thread Face Lift Technique in Patients with Facial Palsy. J. Clin. Aesthet. Dermatol..

[25] Boehm L.M., Morgan A., Hettinger P., Matloub H.S. (2021). Facial Aging: A Quantitative Analysis of Midface Volume Changes over 11 Years. Plast. Reconstr. Surg..

[26] Mertens A., Foyatier J.L., Mojallal A. (2016). Quantitative analysis of midface fat compartments mass with ageing and body mass index, anatomical study. Ann. Chir. Plast. Esthét..

[27] Sarigul Guduk S., Cevik Cenkeri H., Derin Cicek E., Kus S. (2022). Evaluation of aging changes of the superficial fat compartments of the midface over time: A computed tomography study. J. Cosmet. Dermatol..

[28] Iblher N., Stark G.B., Penna V. (2012). The aging perioral region—Do we really know what is happening. J. Nutr. Health Aging.

[29] Wollina U. (2013). Perioral rejuvenation: Restoration of attractiveness in aging females by minimally invasive procedures. Clin. Interv. Aging.

[30] Stutman R.L., Codner M.A. (2012). Tear Trough Deformity: Review of Anatomy and Treatment Options. Aesthetic Surg. J..

[31] Tao B.K., Butt F.R., Dhivagaran T., Balas M., Nijhawan N., Nassrallah G., Hussain A., Ing E.B. (2025). Periocular Aging Across Populations and Esthetic Considerations: A Narrative Review. J. Clin. Med..

[32] Russel S.M., Clark J.M. (2023). Periorbital rejuvenation in the clinic: A state-of-the-art review. World J. Otorhinolaryngol. Head Neck Surg..

[33] Farkas J.P., Pessa J.E., Hubbard B., Rohrich R.J. (2013). The Science and Theory behind Facial Aging. Plast. Reconstr. Surg. Glob. Open.

[34] Swift A., Liew S., Weinkle S., Garcia J.K., Silberberg M.B. (2021). The Facial Aging Process From the "Inside Out". Aesthet. Surg. J..

[35] Minelli L., Yang H.-M., van der Lei B., Mendelson B. (2023). The Surgical Anatomy of the Jowl and the Mandibular Ligament Reassessed. Aesthetic Plast. Surg..

[36] Kapoor K.M., Saputra D.I., Porter C.E., Colucci L., Stone C., Brenninkmeijer E.E.A., Sloane J., Sayed K., Winaya K.K., Bertossi D. (2021). Treating Aging Changes of Facial Anatomical Layers with Hyaluronic Acid Fillers. Clin. Cosmet. Investig. Dermatol..

[37] Kim J., Lee H.R., Jeong J.H., Lee W.S. (2010). Features of facial asymmetry following incomplete recovery from facial paralysis. Yonsei Med. J..

[38] Guntinas-Lichius O., Prengel J., Cohen O., Mäkitie A.A., Vander Poorten V., Ronen O., Shaha A., Ferlito A. (2022). Pathogenesis, diagnosis and therapy of facial synkinesis: A systematic review and clinical practice recommendations by the international head and neck scientific group. Front. Neurol..

[39] Webb B.D., Manoli I., Engle E.C., Jabs E.W. (2021). A framework for the evaluation of patients with congenital facial weakness. Orphanet J. Rare Dis..

[40] Jeong K.Y., Min K.J., Woo J., Yim S.Y. (2015). Craniofacial Asymmetry in Adults With Neglected Congenital Muscular Torticollis. Ann. Rehabil. Med..

[41] Meghe S.R., Khan A., Jangid S.D., Sarda B., Vangala N., Saoji V. (2024). Shedding Light on Acne Scars: A Comprehensive Review of CO_2_ vs. Erbium-Doped Yttrium Aluminium Garnet (Er:YAG) Laser Therapy. Cureus.

[42] Meghe S., Saoji V., Madke B., Singh A. (2024). Efficacy of Microneedling and CO_2_ Laser for Acne Scar Remodelling: A Comprehensive Review. Cureus.

[43] Chaudhary D.C., Kaur S., Bagga D.S., Sharma V., Deshmukh A. (2015). Rarest muscular imbalance, neutral zone shift and facial asymmetry. Med. J. Armed Forces India.

[44] Wollina U., Kocic H., Goldman A. (2023). Hyaluronic Acid in Facial Rehabilitation—A Narrative Review. Cosmetics.

[45] Juhász M.L.W., Levin M.K., Marmur E.S. (2017). The Kinetics of Reversible Hyaluronic Acid Filler Injection Treated With Hyaluronidase. Dermatol. Surg..

[46] Kroumpouzos G., Treacy P. (2024). Hyaluronidase for Dermal Filler Complications: Review of Applications and Dosage Recommendations. JMIR Dermatol..

[47] Cabral L.R.B., Teixeira L.N., Gimenez R.P., Demasi A.P.D., de Brito Junior R.B., de Araújo V.C., Martinez E.F. (2020). Effect of Hyaluronic Acid and Poly-L-Lactic Acid Dermal Fillers on Collagen Synthesis: An in vitro and in vivo Study. Clin. Cosmet. Investig. Dermatol..

[48] Attenello N.H., Maas C.S. (2015). Injectable fillers: Review of material and properties. Facial Plast. Surg..

[49] Fundarò S.P., Salti G., Malgapo D.M.H., Innocenti S. (2022). The Rheology and Physicochemical Characteristics of Hyaluronic Acid Fillers: Their Clinical Implications. Int. J. Mol. Sci..

[50] da Costa A., Biccigo D.G.Z., de Souza Weimann E.T., Mercadante L.M., Oliveira P.R.G., Prebianchi S.B., Abdalla B.M.Z. (2017). Durability of Three Different Types of Hyaluronic Acid Fillers in Skin: Are There Differences Among Biphasic, Monophasic Monodensified, and Monophasic Polydensified Products?. Aesthetic Surg. J..

[51] Salti G., Siquier-Dameto G., Rharbaoui S., Hernandez Malgapo D.M., Innocenti S., Manni M. (2023). An Interim 6-Month Analysis of the Dermatologic Effects and Midface Volume Correction With XTR CL Filler in a Prospective, Single-Center Study. Dermatol. Surg..

[52] Owczarczyk-Saczonek A., Zdanowska N., Wygonowska E., Placek W. (2021). The Immunogenicity of Hyaluronic Fillers and Its Consequences. Clin. Cosmet. Investig. Dermatol..

[53] Kaufman-Janette J., Taylor S.C., Cox S.E., Weinkle S.H., Smith S., Kinney B.M. (2019). Efficacy and safety of a new resilient hyaluronic acid dermal filler, in the correction of moderate-to-severe nasolabial folds: A 64-week, prospective, multicenter, controlled, randomized, double-blind and within-subject study. J. Cosmet. Dermatol..

[54] Jacovella P.F. (2008). Use of calcium hydroxylapatite (Radiesse) for facial augmentation. Clin. Interv. Aging.

[55] Radilla-Flores M.d.C., Márquez-Gutiérrez E.A., Vélez-Palafox M., Castrejón-Vázquez M.I., Chávez-Flores O.C., Chopin-Doroteo M., González-Torres M. (2025). Feasibility of calcium hydroxyapatite (Radiesse^®^) for improving the biomechanical properties of facial burn scars: A pilot study. JPRAS Open.

[56] Loghem J.V., Yutskovskaya Y.A., Philip Werschler W. (2015). Calcium hydroxylapatite: Over a decade of clinical experience. J. Clin. Aesthet. Dermatol..

[57] Su D., Yang W., He T., Wu J., Zou M., Liu X., Li R., Wang S., Lai C., Wang J. (2024). Clinical applications of a novel poly-L-lactic acid microsphere and hyaluronic acid suspension for facial depression filling and rejuvenation. J. Cosmet. Dermatol..

[58] Avelar L.E., Nabhani S., Wüst S. (2025). Unveiling the Mechanism: Injectable Poly-L-Lactic Acid’s Evolving Role-Insights From Recent Studies. J. Cosmet. Dermatol..

[59] Ao Y.J., Yi Y., Wu G.H. (2024). Application of PLLA (Poly-L-Lactic acid) for rejuvenation and reproduction of facial cutaneous tissue in aesthetics: A review. Medicine.

[60] Ditre C.M. (2009). Facial aesthetic correction with injectable poly-L-lactic Acid following removal of malar cheek implants. J. Clin. Aesthet. Dermatol..

[61] Rivkin A. (2022). PMMA-collagen Gel in Nonsurgical Rhinoplasty Defects: A Methodological Overview and 15-year Experience. Plast. Reconstr. Surg. Glob. Open.

[62] Limongi R.M., Tao J., Borba A., Pereira F., Pimentel A.R., Akaishi P., Velasco e Cruz A.A. (2015). Complications and Management of Polymethylmethacrylate (PMMA) Injections to the Midface. Aesthetic Surg. J..

[63] Park T.H., Seo S.W., Kim J.K., Chang C.H. (2012). Clinical experience with polymethylmethacrylate microsphere filler complications. Aesthetic Plast. Surg..

[64] Clark N.W., Pan D.R., Barrett D.M. (2023). Facial fillers: Relevant anatomy, injection techniques, and complications. World J. Otorhinolaryngol. Head Neck Surg..

[65] Funt D., Pavicic T. (2013). Dermal fillers in aesthetics: An overview of adverse events and treatment approaches. Clin. Cosmet. Investig. Dermatol..

[66] Biesman B.S., Green J.B., George R., Jacob C., Palm M., Jones D.H., Grunebaum L., Beer K., Cho Y., Joseph J.H. (2024). A Multicenter, Randomized, Evaluator-Blinded Study to Examine the Safety and Effectiveness of Hyaluronic Acid Filler in the Correction of Infraorbital Hollows. Aesthet. Surg. J..

[67] Bhojani-Lynch T., Deckers A., Ohanes O., Poupard K., Maffert P. (2021). A Prospective, Observational Registry Study to Evaluate Effectiveness and Safety of Hyaluronic Acid-Based Dermal Fillers in Routine Practice: Interim Analysis Results with One Year of Subject Follow-Up. Clin. Cosmet. Investig. Dermatol..

[68] Lee J.M., Kim Y.J. (2015). Foreign body granulomas after the use of dermal fillers: Pathophysiology, clinical appearance, histologic features, and treatment. Arch. Plast. Surg..

[69] Hong G.W., Hu H., Chang K., Park Y., Lee K.W.A., Chan L.K.W., Yi K.H. (2024). Review of the Adverse Effects Associated with Dermal Filler Treatments: Part I Nodules, Granuloma, and Migration. Diagnostics.

[70] Al-Zahawi S., Ehsani A., Jozdani T., Rahimnia A., Ehsani A.H., Razavi Z., Emadi S.N. (2025). The Demographics of Patients With Dermal Filler Complications. J. Cosmet. Dermatol..

[71] Dierckx S., Patrizi M., Merino M., González S., Mullor J.L., Nergiz-Unal R. (2024). Collagen peptides affect collagen synthesis and the expression of collagen, elastin, and versican genes in cultured human dermal fibroblasts. Front. Med..

[72] Bernardo R.T.R., Oliveira R.C.G., Freitas K.M.S., Albergaria-Barbosa J.R., Rizzatti-Barbosa C.M. (2024). Effect of poly-L-lactic acid and polydioxanone biostimulators on type I and III collagen biosynthesis. Skin Res. Technol..

[73] Kim C.M., Kim B.Y., Hye Suh D., Lee S.J., Moon H.R., Ryu H.J. (2019). The efficacy of powdered polydioxanone in terms of collagen production compared with poly-L-lactic acid in a murine model. J. Cosmet. Dermatol..

[74] Zhou S.Y., Kang S.M., Gu Y.J., Zhang X.R., Yon D.K., Shin B.H., Ham J.R., Lee W.K., Jeong J.G., Kwon H.J. (2023). Bio-characteristics and Efficacy Analysis of Biodegradable Poly Dioxanone Dermal Filler in a Mouse Model and Humans. In Vivo.

[75] Sedush N.G., Kalinin K.T., Azarkevich P.N., Gorskaya A.A. (2023). Physicochemical Characteristics and Hydrolytic Degradation of Polylactic Acid Dermal Fillers: A Comparative Study. Cosmetics.

[76] Bolke L., Schlippe G., Gerß J., Voss W. (2019). A Collagen Supplement Improves Skin Hydration, Elasticity, Roughness, and Density: Results of a Randomized, Placebo-Controlled, Blind Study. Nutrients.

[77] Trehan A., Anand R., Chaudhary G., Garg H., Verma M.K. (2024). Efficacy and Safety of Skin Radiance Collagen on Skin and Hair Matrix: A Placebo-Controlled Clinical Trial in Healthy Human Subjects. Clin. Cosmet. Investig. Dermatol..

[78] Hong G.W., Kim S.B., Park S.Y., Wan J., Yi K.H. (2024). Thread Lifting Materials: A Review of Its Difference in Terms of Technical and Mechanical Perspective. Clin. Cosmet. Investig. Dermatol..

[79] Giang N.N., Kim H.J., Chien P.N., Kwon H.J., Ham J.R., Lee W.K., Gu Y.J., Zhou S.Y., Zhang X.R., Nam S.Y. (2024). An evaluation of the effectiveness of ‘ULTRACOL 200’ in enhancing nasolabial fold wrinkles through cutaneous repair. Skin Res. Technol..

[80] Su D., Wang S., He T., Wang J. (2024). Experimental investigation of biostimulatory effects after polydioxanone thread insertion in a pig model. J. Cosmet. Dermatol..

[81] Sulyman O., Cristel R., Gandhi N., Kola E., Borst S.G., Caughlin B., Dayan S. (2024). Non-surgical rhinoplasty using polydioxanone threads. J. Cosmet. Dermatol..

[82] Unal M., İslamoğlu G.K., Ürün Unal G., Köylü N. (2021). Experiences of barbed polydioxanone (PDO) cog thread for facial rejuvenation and our technique to prevent thread migration. J. Dermatol. Treat..

[83] Ali Y.H. (2018). Two years’ outcome of thread lifting with absorbable barbed PDO threads: Innovative score for objective and subjective assessment. J. Cosmet. Laser Ther..

[84] Niu Z., Han Y., Jin R., Li Y., Liu J., Li N., Li W., Li D., Chen Y., Han Y. (2020). Complications Following Facial Thread-Lifting. Chin. J. Plast. Reconstr. Surg..

[85] Contreras C., Ariza-Donado A., Ariza-Fontalvo A. (2023). Using PDO threads: A scarcely studied rejuvenation technique. Case report and systematic review. J. Cosmet. Dermatol..

[86] Zhukova O., Dydykin S., Kubíková E., Markova N., Vasil’ev Y., Kapitonova M. (2022). A New Complex Minimally Invasive Thread Lift Method for One-Time Three-Step Fixation of the Face and Neck Soft Tissues. Arch. Plast. Surg..

[87] Burko P., Sulamanidze G., Nikishin D. (2025). Efficacy of Lifting Threads Composed of Poly(L-Lactide-Co-ε-Caprolactone) Copolymers Coated With Hyaluronic Acid: A Long-Term Study on Biorevitalizing Properties in Skin Remodeling. J. Cosmet. Dermatol..

[88] Cho S.W., Shin B.H., Heo C.Y., Shim J.H. (2021). Efficacy study of the new polycaprolactone thread compared with other commercialized threads in a murine model. J. Cosmet. Dermatol..

[89] Hügül H., Özkoca D., Kutlubay Z. (2022). Thread lifting: Does patient satisfaction change according to age, type of threads used, number of threads used and treatment area?. J. Cosmet. Dermatol..

[90] Middleton E.O., Karypidis D. (2023). Validation of Non-surgical Facial Lifting with PDO Thread using a 3D system. Adv. Oral Maxillofac. Surg..

[91] Ehlinger-David A., Gorj M., Braccini F., Loreto F., Grand-Vincent A., Garcia P., Taieb M., Benadiba L., Catoni I., Mathey E.R. (2023). A prospective multicenter clinical trial evaluating the efficacy and safety of a hyaluronic acid-based filler with Tri-Hyal technology in the treatment of lips and the perioral area. J. Cosmet. Dermatol..

[92] Yi K.H., Park S.Y. (2025). Facial Thread Lifting Complications. J. Cosmet. Dermatol..

[93] Alster T.S., Lupton J.R. (2007). Nonablative cutaneous remodeling using radiofrequency devices. Clin. Dermatol..

[94] Byun J.W., Kang Y.R., Park S., Hong W. (2023). Efficacy of radiofrequency combined with single-dot ultrasound efficacy for skin rejuvenation: A non-randomized split-face trial with blinded response evaluation. Skin Res. Technol..

[95] Dayan E., Theodorou S. (2022). Not all Radiofrequency Devices Are Created Equal: A Thermal Assessment. Plast. Reconstr. Surg. Glob. Open.

[96] el-Domyati M., el-Ammawi T.S., Medhat W., Moawad O., Brennan D., Mahoney M.G., Uitto J. (2011). Radiofrequency facial rejuvenation: Evidence-based effect. J. Am. Acad. Dermatol..

[97] Oh S., Rhee D.Y., Batsukh S., Son K.H., Byun K. (2023). High-Intensity Focused Ultrasound Increases Collagen and Elastin Fiber Synthesis by Modulating Caveolin-1 in Aging Skin. Cells.

[98] Park H., Kim E., Kim J., Ro Y., Ko J. (2015). High-Intensity Focused Ultrasound for the Treatment of Wrinkles and Skin Laxity in Seven Different Facial Areas. Ann. Dermatol..

[99] Guo H., Zhang X., Li H., Fu C., Jiang L., Hu Y., Huang J., Chen J., Zeng Q. (2023). Dynamic panoramic presentation of skin function after fractional CO(2) laser treatment. iScience.

[100] Preissig J., Hamilton K., Markus R. (2012). Current Laser Resurfacing Technologies: A Review that Delves Beneath the Surface. Semin. Plast. Surg..

[101] Shin J., Sung Y., Jin S., Hwang C.-L., Kim H., Hong D., Jung K.E., Seo Y.-J., Lee Y. (2024). Efficacy and Safety of Monopolar Radiofrequency for Tightening the Skin of Aged Faces. Cosmetics.

[102] Werschler W.P., Werschler P.S. (2016). Long-term Efficacy of Micro-focused Ultrasound with Visualization for Lifting and Tightening Lax Facial and Neck Skin Using a Customized Vectoring Treatment Method. J. Clin. Aesthet. Dermatol..

[103] Meaike J.D., Agrawal N., Chang D., Lee E.I., Nigro M.G. (2016). Noninvasive Facial Rejuvenation. Part 3: Physician-Directed-Lasers, Chemical Peels, and Other Noninvasive Modalities. Semin. Plast. Surg..

[104] Piet A., Jablonski L., Daniel Onwuchekwa J.I., Unkel S., Weber C., Grzegorzek M., Ehlers J.P., Gaus O., Neumann T. (2023). Non-Invasive Wearable Devices for Monitoring Vital Signs in Patients with Type 2 Diabetes Mellitus: A Systematic Review. Bioengineering.

[105] Shi Y., Wu W. (2023). Multimodal non-invasive non-pharmacological therapies for chronic pain: Mechanisms and progress. BMC Med..

[106] Mysore V., Deepthi M., Chandrashekar B.S., Shah S.D., Gold M.H., Shivani S.R., Kanumuru P., Anirudh P. (2024). Standard operating protocol for utilizing energy-based devices in aesthetic practice. J. Cosmet. Dermatol..

[107] Haykal D. (2024). Emerging and Pioneering AI Technologies in Aesthetic Dermatology: Sketching a Path Toward Personalized, Predictive, and Proactive Care. Cosmetics.

[108] Li C., Li Z., Shi L., Wang P., Gao F., Sun W. (2021). Effectiveness of Focused Shockwave Therapy versus Radial Shockwave Therapy for Noncalcific Rotator Cuff Tendinopathies: A Randomized Clinical Trial. Biomed. Res. Int..

[109] Ko N.Y., Chang C.N., Cheng C.H., Yu H.K., Hu G.C. (2022). Comparative Effectiveness of Focused Extracorporeal versus Radial Extracorporeal Shockwave Therapy for Knee Osteoarthritis-Randomized Controlled Study. Int. J. Environ. Res. Public Health.

[110] Alshihri A., Kämmerer P.W., Heimes D., Niu W., Alnassar T., Spector M. (2020). Extracorporeal Shock Wave Stimulates Angiogenesis and Collagen Production in Facial Soft Tissue. J. Surg. Res..

[111] Simplicio C.L., Purita J., Murrell W., Santos G.S., Dos Santos R.G., Lana J. (2020). Extracorporeal shock wave therapy mechanisms in musculoskeletal regenerative medicine. J. Clin. Orthop. Trauma.

[112] Lv F., Li Z., Jing Y., Sun L., Li Z., Duan H. (2023). The effects and underlying mechanism of extracorporeal shockwave therapy on fracture healing. Front. Endocrinol..

[113] Sukubo N.G., Tibalt E., Respizzi S., Locati M., d’Agostino M.C. (2015). Effect of shock waves on macrophages: A possible role in tissue regeneration and remodeling. Int. J. Surg..

[114] Kou D., Chen Q., Wang Y., Xu G., Lei M., Tang X., Ni H., Zhang F. (2024). The application of extracorporeal shock wave therapy on stem cells therapy to treat various diseases. Stem Cell Res. Ther..

[115] Chen Y., Lyu K., Lu J., Jiang L., Zhu B., Liu X., Li Y., Liu X., Long L., Wang X. (2022). Biological response of extracorporeal shock wave therapy to tendinopathy in vivo (review). Front. Vet. Sci..

[116] Ko J., Cho S.B. (2024). Clinical Efficacy and Safety of Low-Energy Extracorporeal Shock Wave Therapy for Various Conditions of Deep Dermal and Subdermal Fibrosis. Skin Res. Technol..

[117] de Lima Morais T.M., Meyer P.F., de Vasconcellos L.S., e Silva J.C., e Andrade I.F., de Farias V.A.F., da Silva I.C., Araújo R.M.F.G., da Silva R.M.V., Pacheco E.F. (2019). Effects of the extracorporeal shock wave therapy on the skin: An experimental study. Lasers Med. Sci..

[118] Kimura K., Tanaka Y. (2021). Facial Tightening Effects, Following Focused and Radial Acoustic Wave Therapy Assessment, Using a Three-Dimensional Digital Imaging. Lasers Surg. Med..

[119] Knobloch K., Kraemer R. (2015). Extracorporeal shock wave therapy (ESWT) for the treatment of cellulite—A current metaanalysis. Int. J. Surg..

[120] Goel A., Rai K. (2022). Non-surgical facelift-by PDO threads and dermal filler: A case report. J. Cosmet. Dermatol..

[121] Levy L.L., Emer J.J. (2012). Complications of minimally invasive cosmetic procedures: Prevention and management. J. Cutan. Aesthet. Surg..

[122] Rahbin S., Sunnergren O., McBride E., Darabi H., Alinasab B. (2024). Does More Invasive Surgery Result in Higher Patient Satisfaction? A Long-Term Follow-Up of 136 Zygomaticomaxillary Complex Fractures. Craniomaxillofac. Trauma Reconstr..

[123] Harb A., Abdul-Razzak A. (2024). Nonsurgical Correction of Surgical Rhinoplasty Complications with Hyaluronic Acid Fillers: A Retrospective Review of 2088 Cases. Plast. Reconstr. Surg. Glob. Open.

[124] Rękas-Dudziak A., Męcińska-Jundziłł K., Walkowiak K., Witmanowski H. (2023). The use of local anaesthetics in dermatology, aesthetic medicine and plastic surgery: Review of the literature. Postep. Dermatol. Alergol..

[125] Smith L., Cockerham K. (2011). Hyaluronic acid dermal fillers: Can adjunctive lidocaine improve patient satisfaction without decreasing efficacy or duration? *Patient Prefer*. Adherence.

[126] Alenazi N.F., AlBattal N.Z., Albalawi I.A.S., Saleh N.A., Alnaim M.F., Mahmoud A.Z.B., Vasilescu D.C. (2024). Rate and Predictors of Satisfaction after Noninvasive Facial Cosmetic Procedures: A National Study in Saudi Arabia. Plast. Reconstr. Surg. Glob. Open.

[127] Cohen J.L., Rivkin A., Dayan S., Shamban A., Werschler W.P., Teller C.F., Kaminer M.S., Sykes J.M., Weinkle S.H., Garcia J.K. (2022). Multimodal Facial Aesthetic Treatment on the Appearance of Aging, Social Confidence, and Psychological Well-being: HARMONY Study. Aesthet. Surg. J..

[128] Nestor M.S. (2019). Facial Lift and Patient Satisfaction Following Treatment with Absorbable Suspension Sutures: 12-Month Data from a Prospective, Masked, Controlled Clinical Study. J. Clin. Aesthet. Dermatol..

